# Sonoelastographic Assessment of the Achilles Tendon in Familial Mediterranean Fever Patients: Comparison With Healthy Subjects

**DOI:** 10.7759/cureus.12143

**Published:** 2020-12-18

**Authors:** Seyfi Evran, Mehtap Beker-Acay, Sinan Saracli, Akif Acay, Emre Kacar, Furkan Kaya

**Affiliations:** 1 Radiology, Afyonkarahisar State Hospital, Afyonkarahisar, TUR; 2 Radiology, Afyonkarahisar Health Sciences University, Afyonkarahisar, TUR; 3 Statistics, Afyon Kocatepe University, Afyonkarahisar, TUR; 4 Internal Medicine, Kutahya Park Hayat Hospital, Kutahya, TUR; 5 Radiology, Doruk Setbasi Medical Center, Bursa, TUR

**Keywords:** sonoelastography, tendinosis, mediterranean fever, familial

## Abstract

Introduction and objective: This study aims at using sonoelastography as a novel technique to evaluate the stiffness and thickness of Achilles tendons in familial Mediterranean fever (FMF) patients.

Methods: Achilles tendons of 26 FMF patients and 23 control subjects were assessed with ultrasound and real-time sonoelastography. The Achilles tendons were divided into the distal, middle, and proximal thirds for elastographic image evaluation. Tendons were classified into three main types according to their elasticity features: grade 1 blue (hardest tissue) to green (hard tissue); grade 2, yellow (soft tissue); and grade 3, red (softest tissue). Tendons of the groups were compared in terms of thickness and stiffness.

Results: There were no significant differences in thickness and stiffness of the Achilles tendon between FMF patients and controls (p>0.05). Sonoelastography of Achilles tendons of FMF patients displayed no relationship between FMF and tendinopathy.

Conclusion: This issue should be explored in prospective studies in larger groups.

## Introduction

The Achilles tendon is the thickest tendon in the human body and serves to attach the plantaris, gastrocnemius, and soleus muscles to the calcaneal tuberosity in the lower leg [1]. Enthesopathy is a specific sign of spondyloarthropathies that occasionally accompany familial Mediterranean fever (FMF). Achilles tendons may be involved as tendinitis or enthesitis and may lead to heel pain and stiffness [2-4]. FMF is one of the foremost reasons for secondary amyloidosis, which continues to be a major cause of systemic amyloidosis. Amyloid deposition may cause synovitis and inflammation [5]. It is a genetically transmitted rheumatic disease also characterized by recurrent abdominal pain, fever, joint pain and swelling attacks [2].

Ultrasonography is a very effective method in the detection and evaluation of Achilles tendonosis and detection of peripheral joint pathologies. After the description of sonoelastography (SE) in 1991, it was implemented in the detection and assessment of various liver diseases, breast and thyroid mass lesions, and lymph node pathologies [6,7]. It is a novel ultrasonography (US) technique that identifies the changes in stiffness of tissues and it can be used to explore the tendon abnormalities before they can be detected by US, so it potentially makes a significant contribution to the diagnostic accuracy in tendinopathy [4,8]. SE propagates a strain in the tissue during compression which is less in hard tissues than in soft tissues, consequently providing an objective definition of tissue stiffness [1]. The Achilles tendon is an ideal structure for elastography examination in terms of thickness and location, and very few studies in the literature concern Achilles tendinopathy in FMF patients. In this study, we aimed at using strain SE as a novel technique to evaluate the stiffness of the Achilles tendon in FMF patients. 

## Materials and methods

Participants and examinations

This prospectively designed study comprised 26 patients with FMF disease (eight male, 18 female) and 23 control subjects (10 male, 13 female). The patient group had been diagnosed and followed up with FMF according to their clinical and laboratory findings in our institution for two to 10 years. The patients’ sonographic evaluations were carried out during routine follow-up visits after physical examination and laboratory analyses. None of the subjects had musculoskeletal complaints concerning the lower extremities. Those with known metabolic or endocrine diseases, sports injury or trauma history, and patients under 18 years of age were excluded from the study. The institutional ethics committee approved the study (protocol number: 2011 KAEK-2, 22 April 2015).

B-mode US and SE examinations were performed in transverse and longitudinal planes using the Hitachi Preirus (Tokyo, Japan) ultrasound device and 13-MHz linear probe. During the examination, the patients were in a prone position with their ankles and feet hanging over the edge of the bed in a relaxed position to avoid tendon stress. Sonographic evaluation of the Achilles tendon was done by one sonographer experienced in tendon imaging. The Achilles tendon was divided into the following thirds: proximal third (musculotendinous junction where the most proximal part of the tendon that can be observed), middle third (mid-level of the calf muscles, 2-6 cm above insertion at the calcaneus), and distal third (insertion at the calcaneus). The thickness of the tendon was determined by measuring the anteroposterior diameter in a transverse view at these levels. Longitudinal and axial images were obtained of each tendon third using US and real-time SE. Besides tendinopathy, any findings related to the tendon such as calcification, degeneration, or pathology of paratendon structures like bone erosions or bursitis were also noted.

**Figure 1:**
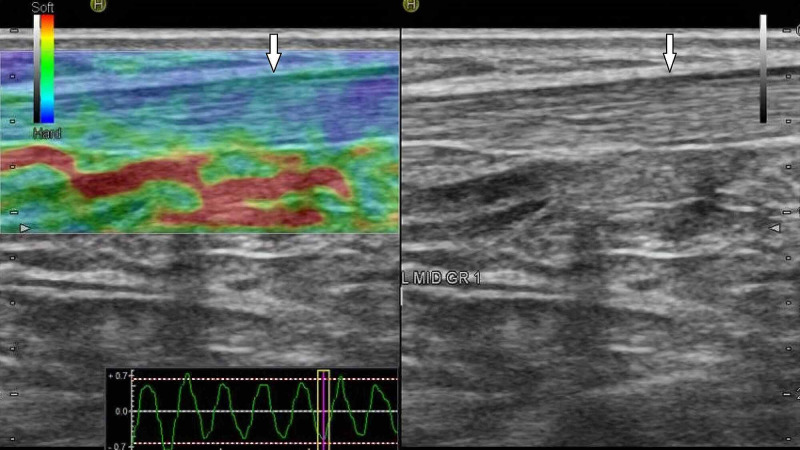
Normally appearing grade 1 Achilles tendon. B-mode (right image) and sonoelastographic (left image) ultrasound images of the left middle part of Achilles tendon (arrows) in a 21-year-old woman with familial Mediterranean fever (FMF).

**Figure 2:**
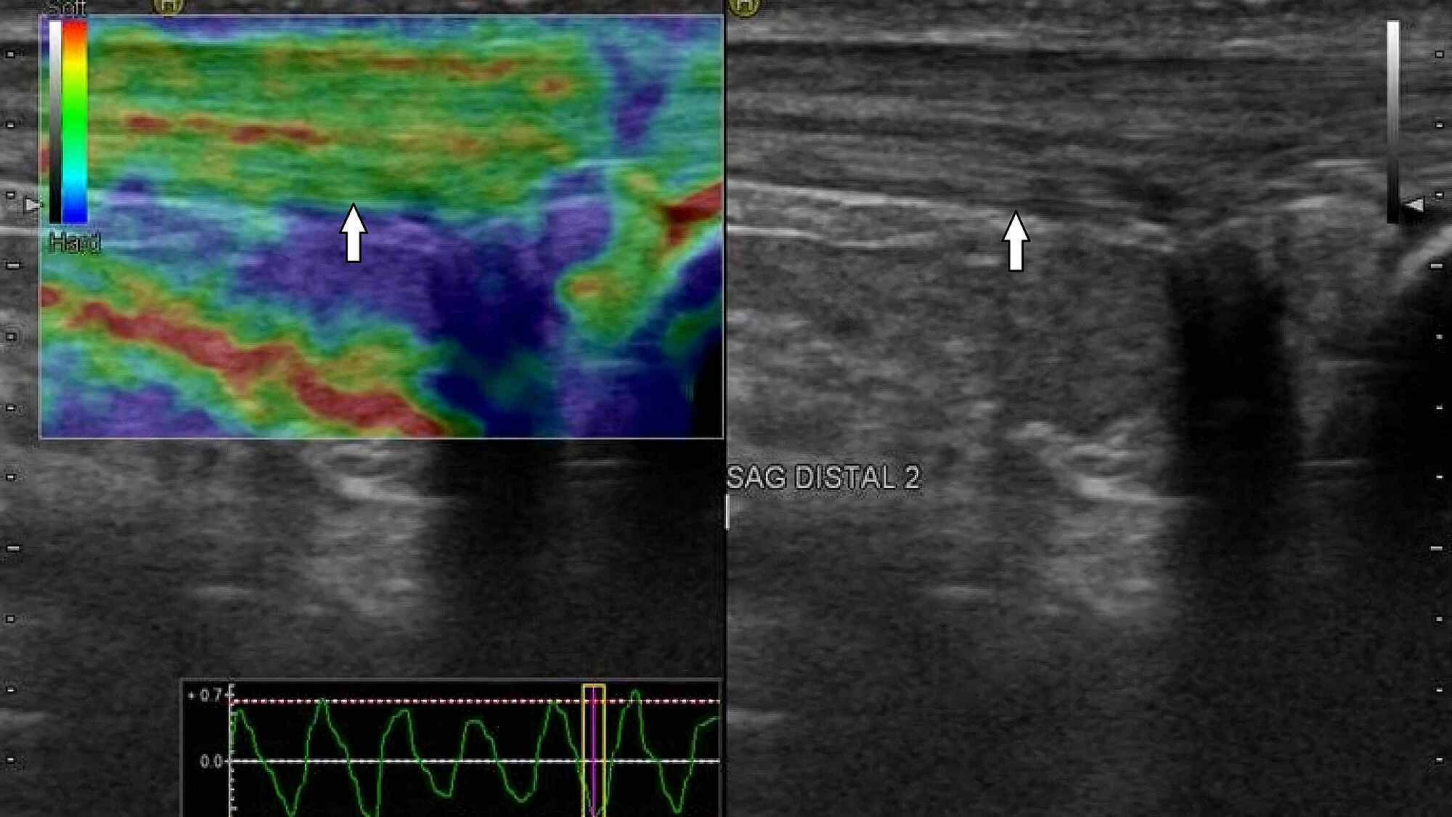
Grade 2 Achilles tendon. B-mode (right image) and sonoelastographic (left image) ultrasound images of the distal part of right Achilles tendon (arrows) in a 46-year-old healthy woman.

**Figure 3:**
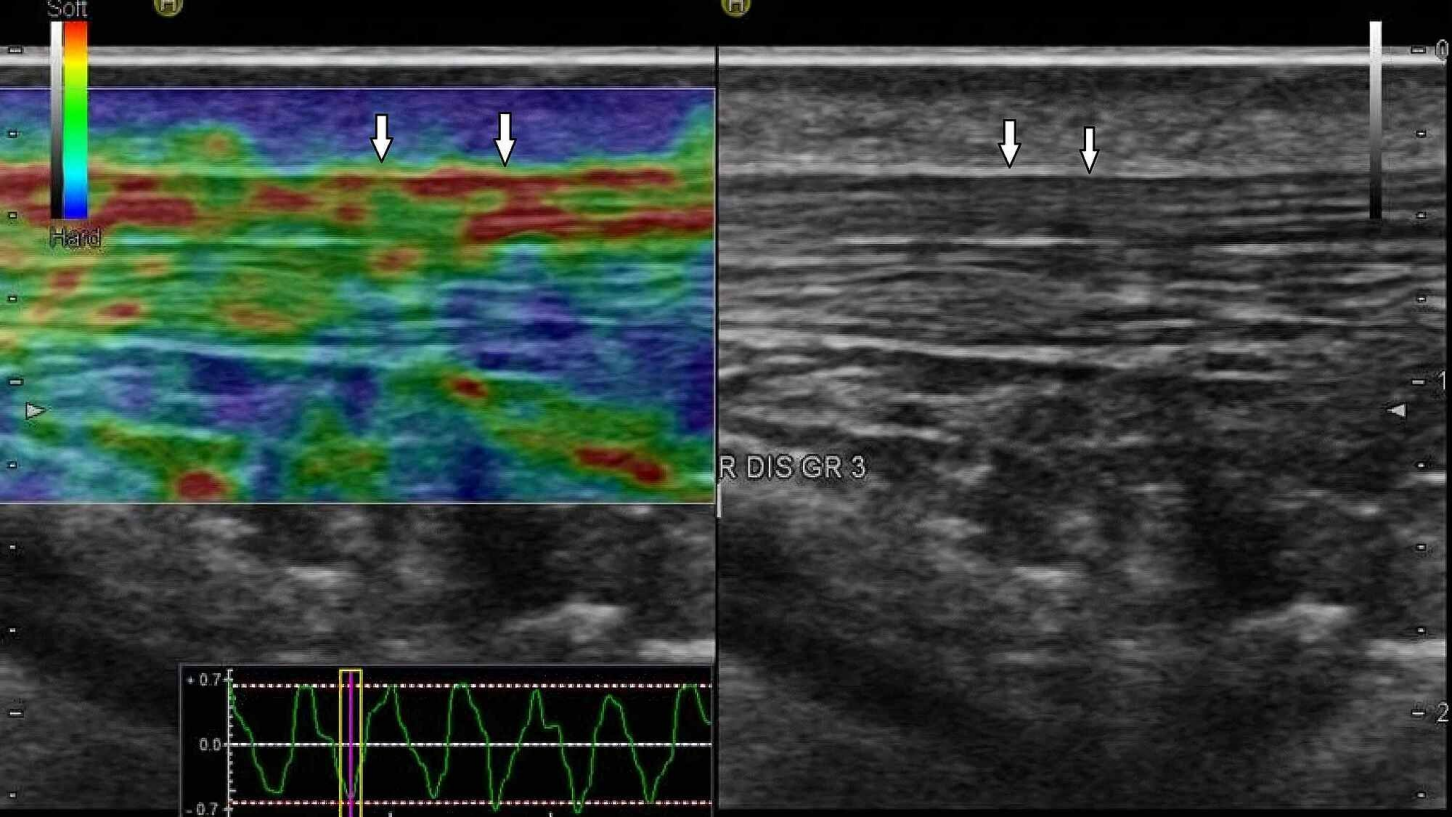
Grade 3 Achilles tendon. B-mode (right image) and sonoelastographic (left image) ultrasound images of the distal part of right Achilles tendon (arrows) in a 32-year-old woman with familial Mediterranean fever (FMF). Structurally impaired  tendon in shades of yellow to red.

SE was performed by repetitive, mild, perpendicular pressure to the proximal, middle, and distal thirds of the Achilles tendon by the same sonographer. With the aim of improving the signal-to-noise ratio of real-time SE images, multicompression technique was applied. By using a freehand technique, local strain was computed under slight compression and decompression. The pressure applied to the Achilles tendon was adjusted according to the visual indicator for compression, which displays the average strain in the region of interest between two frames and shows optimal strain on the screen. With the aim of standardizing the SE color gathering and image quality a color bar on the ultrasound screen was located. The Achilles tendon was evaluated according to the classification of De Zordo et al. [6] as follows: grade 1, blue (hardest tissue) to green (hard tissue); grade 2, yellow (soft tissue); and grade 3, red (softest tissue) (Figures 1-3).

Statistical analysis

Statistical Package for Social Sciences (SPSS; IBM Corp., Armonk, NY, USA) was used for statistical analysis. The descriptive data were stated as mean, median, standard deviation, number, and percentage. In the comparison of tendon stiffness grade and thickness of the patient and control groups, the Student t-test was applied. Categorical variables were compared by the χ2 test. P < 0.05 was considered statistically significant.

## Results

The mean age of the patients was 42.03 years (range, 18-70 years) and the mean age for the control group was 44.4 years (range, 28-60 years).

Unilateral calcification in the distal insertion of the tendon was found in 10 subjects in the patient group and 16 subjects in the control group. No other paratendinous structure pathologies was observed.

No statistical difference in regard to the tendon thickness measurements of FMF patients and the control group was found (p>0.05) (Table 1).

**Table 1:** Tendon thickness measurements of the subjects (mm).

	FMF	Control	P value
Left			
Proximal	4.06	4.11	0.8
Middle	4.81	4.99	0.34
Distal	5.20	5.68	0.19
Right			
Proximal	3.91	4.17	0.17
Middle	4.90	5.41	0.58
Distal	6.44	6.77	0.47

In terms of SE findings, no statistical difference was found between FMF patients and the control group (p>0.05) (Table 2).

**Table 2:** Real-time sonoelastography findings in familial Mediterranean fever (FMF) patients and healthy subjects. Number of tendon thirds (%).

	Right			Left			Total (n)
	Distal	Middle	Proximal	Distal	Middle	Proximal	
FMF Group							
Grade 1	5 (19.2)	7 (26.9)	11 (42.3)	7 (26.9)	13 (50)	18 (69.2)	61
Grade 2	15 (57.7)	14 (53.8)	11 (42.3)	14 (53.8)	12 (46.2)	5 (19.2)	71
Grade 3	6 (23.1)	5 (19.2)	4 (15.4)	5 (19.2)	1 (3.8)	3 (11.5)	24
Control Group							
Grade 1	6 (26.1)	9 (39.1)	12 (52.2)	3 (13)	11 (47.8)	11 (47.8)	52
Grade 2	12 (52.2)	14 (60.9)	10 (43.5)	16 (69.6)	10 (43.5)	11 (47.8)	73
Grade 3	5 (21.7)	0	1 (4.3)	4 (17.4)	2 (8.7)	1 (4.3)	13
P value	0.33	0.67	0.10	0.49	0.76	0.25	

## Discussion

The link between tendinopathy and FMF has been a debate. Some studies concluded that there is a link between enthesopathy and FMF [2,3,5]. The most striking finding of our study was no statistically significant difference was reached in terms of tendon thickness or SE grading of Achilles tendon enthesopathy. Although enthesopathy was evaluated by gray scale and Doppler US findings, SE examination was rarely performed to date. This technique was introduced as a novel method to display early histopathologic changes in Achilles tendinosis [9].

In the study by Ozkan et al., the authors used Madrid Sonographic Enthesitis Index (MASEI) including three elemental lesions: calcification, Doppler US, and erosion, to evaluate the tendon structure, in addition to tendon thickness, bursa, and upper limb examinations. They compared a patient group of 50 FMF patients with 57 healthy subjects and found that the frequency of entheseal abnormalities was significantly increased in patients with FMF. On the other hand, no difference was observed between patients’ and controls’ MASEI scores and disease duration or colchicine treatment duration. Additionally, no difference was observed between the MASEI score and the presence or absence of arthritic involvement among the patients [5].

In a previous SE study Achilles tendon abnormalities in patients with FMF suffering from heel pain were evaluated. The authors compared Achilles tendons of 18 FMF patients suffering from unilateral heel pain with the normal side. Achilles tendon thicknesses were measured at three segments and no statistically significant difference was observed between the painless and symptomatic sides. The proximal part of the Achilles tendon’s elasticity was not statistically different in both sides (p=0.31). A statistical difference was observed in the middle and distal segments, in regard to the elasticity in the symptomatic sides than normal sides (p=0.005 and p=0.001 respectively) [3]. We constructed our study independently of achillodynia or heel pain in order not to cause any bias as we aimed to explore if there is any link between the inflammatory process and tendinopathy.

According to a retrospective study of 33 patients, the outcome of achillodynia may be predicted by the grade assigned to the Achilles tendon’s appearance on sonograms. This possible use of tendon US may be implemented as a prognostic tool to supplement physical examination [10]. In the study by Tufan et al., the prevalence of enthesopathy in FMF patients was found to be similar to that observed in healthy subjects and this outcome of the study resembles our results. They found that enthesopathy was higher in the FMF-associated spondyloarthropathy group and almost all patients of this group had the M694V mutation. These observations suggest that not the FMF phenotype itself, but the M694V mutation is associated with the enthesopathy in these patients, which is the hallmark and the possible major process in the pathogenesis of spondyloarthropathies [11].

In the prospective case-control study by Ciloglu et al., using SE, they evaluated the healing process of a torn Achilles tendon after surgical repair. They found that the Achilles tendon seems to become stiffer with the healing process. SE can provide structural information about the healing process of the Achilles tendon after surgical repair and can quantify findings for follow-up [12]. In the current study no significant changes were found in the thickness or SE grades of the Achilles tendon. Although US is highly sensitive in detecting the inflammatory manifestations of acute and chronic enthesitis, it is not successful in showing early changes in the mechanical properties of the tendon. SE has been introduced to provide qualitative and quantitative stiffness measurements that reveal early alterations in the mechanical properties of tissues [4].

In a study comprising 32 patients with FMF, 31 with Behçet’s Disease (BD), and 35 control subjects, there was no difference in terms of tendon thickness measurements of FMF patients when compared with the other groups, even with the existence of arthritic involvement. Both FMF and BD groups' tendon thicknesses were found to be positively correlated with height and body weight of the subjects. Either in FMF or BD groups, disease duration didn't correlate with the tendon thicknesses. Furthermore, no correlation with the tendon measurements was found in FMF patients with regard to colchicine treatment duration or cumulative number of attacks [2].

Zardi et al. compared the Achilles tendon stiffness and thickness of the patients affected by ankylosing spondylitis (AS) who were treated with anti-TNF-α for two years with controls. They evaluated 22 Achilles tendons of 11 AS patients and 26 of 13 controls and found no significant differences in these parameters between the groups, except for an increased thickness in the middle third of the tendon in the AS patients (p=0.04) [4].

A limitation of this study was that the groups were not matched for body mass index, height, or weight, factors that might influence the enthesis score. We designed our study regardless of the duration of the disease and we did not record the duration of colchicine treatment. Shear wave elastography (SWE) is considered more objective than SE, as it is less influenced by inter-operator variability, providing potentially more reproducible results than compression SE. Also, it allows the assessment of qualitative elastograms and quantitative measurements. Although SE is a semi-quantitative technique some limitations regarding SWE must be considered, particularly the limited size, shape, and depth of the region of interest (ROI). There have been conflicting results regarding the reproducibility of using shear wave elastography to assess the stiffness of the tendon, probably due to the fiber structure of the tissue [4,13]. These findings should be confirmed with MRI and pathologic evaluation should be established in future studies in larger groups.

## Conclusions

In conclusion, this study showed no difference in tendon stiffness of FMF patients compared to control subjects, regardless of disease duration. We believe that this issue should be addressed with prospective studies in larger groups.

## References

[1] Tan S, Kudaş S, Özcan AS, İpek A, Karaoğlanoğlu M, Arslan H, Bozkurt M (2012). Real-time sonoelastography of the Achilles tendon: pattern description in healthy subjects and patients with surgically repaired complete ruptures. Skeletal Radiol.

[2] Ozçakar L, Onat AM, Ureten K (2006). Sonographic evaluation of the tendons in familial Mediterranean fever and Behçet's disease. Joint Bone Spine.

[3] Yakut ZI, Oğur T, Erten S, Delibaş D, Yıldırım M, Arslan H, Gümüş M (2015). Early diagnosis of tendon pathologies with sonoelastography. TAF Prev Med Bull.

[4] Zardi EM, Pipita ME, Giorgi C, Afeltra A, Maffulli N, Franceschi F (2019). Strain ultrasound elastography in the Achilles tendon of ankylosing spondylitis patients treated with anti-TNF-α: a preliminary study. In Vivo.

[5] Ozkan F, Cetin GY, Inci MF, Bakan B, Yuksel M, Ekerbicer HC, Sayarlioglu M (2013). Increased enthesopathy in patients with familial Mediterranean fever: evaluation with a new sonographic enthesitis index. J Ultrasound Med.

[6] De Zordo T, Fink C, Feuchtner GM, Smekal V, Reindl M, Klauser AS (2009). Real-time sonoelastography findings in healthy Achilles tendons. AJR Am J Roentgenol.

[7] Turan A, Tufan A, Mercan R (2013). Real-time sonoelastography of Achilles tendon in patients with ankylosing spondylitis. Skeletal Radiol.

[8] Prado-Costa R, Rebelo J, Monteiro-Barroso J, Preto AS (2018). Ultrasound elastography: compression elastography and shear-wave elastography in the assessment of tendon injury. Insights Imaging.

[9] Klauser AS, Miyamoto H, Tamegger M (2013). Achilles tendon assessed with sonoelastography: histologic agreement. Radiology.

[10] Archambault JM, Wiley JP, Bray RC, Verhoef M, Wiseman DA, Elliott PD (1998). Can sonography predict the outcome in patients with achillodynia?. J Clin Ultrasound.

[11] Tufan A, Mercan R, Tezcan ME (2013). Enthesopathy in patients with familial Mediterranean fever: increased prevalence in M694 V variant. Rheumatol Int.

[12] Ciloglu O, Görgülü FF (2020). Evaluation of a torn Achilles tendon after surgical repair: an ultrasound and elastographic study with 1-year follow-up. J Ultrasound Med.

[13] Ooi CC, Malliaras P, Schneider ME, Connell DA (2014). "Soft, hard, or just right?" Applications and limitations of axial-strain sonoelastography and shear-wave elastography in the assessment of tendon injuries. Skeletal Radiol.

